# Ethnopharmacological survey of medicinal plants used by patients with psoriasis in the West Bank of Palestine

**DOI:** 10.1186/s12906-016-1503-4

**Published:** 2017-01-03

**Authors:** Ramzi Shawahna, Nidal Amin Jaradat

**Affiliations:** 1 Department of Physiology and Pharmacology, Faculty of Medicine and Health Sciences, An-Najah National University, New Campus, Building: 19, Office: 1340, PO Box 7, Nablus, Palestine; 2 An-Najah BioSciences Unit, Center for Poisons Control, Chemical and Biological Analyses, An-Najah National University, Nablus, Palestine; 3 Department of Pharmacy, Faculty of Medicine and Health Sciences, An-Najah National University, Nablus, Palestine

**Keywords:** Ethnopharmacology, Psoriasis, Medicinal plants, CAM, Palestine

## Abstract

**Background:**

Psoriasis is a frequent skin inflammatory disorder that inflicts millions of patients around the globe. To meet their healthcare needs, patients with psoriasis often seek treatment outside the allopathic paradigm. Use of medicinal plants has emerged as one of the most common and preferred modalities of complementary and alternative medicine (CAM). The aim of this study was to investigate the use of medicinal plants by patients with psoriasis in the West Bank of Palestine.

**Methods:**

The current study was a questionnaire based cross-sectional descriptive study on the use of medicinal plants by psoriasis patients in the West Bank of Palestine. A sample of 149 patients with psoriasis who were visiting outpatient clinics responded to the questionnaire in face to face interviews.

**Results:**

Medicinal plants were used by 81 (54.4%) patients with psoriasis. Patients used 33 medicinal plants belonging to 26 families. Plants belonging to Lamiaceae and Leguminosae were the most commonly used by the study patients. *Aloe vera*, *Trigonella arabica*, *Catharanthus roseus* and *Anthemis cotula* were the most frequently used medicinal plants to treat psoriasis. Leaves and fruits were the most commonly used parts by the study patients. Paste was the most commonly used form of preparation. The use of medicinal plants was significantly associated with age and monthly household income of the patients. Enhancement of immunity, improving conventional therapy and reduction of side effects were the most commonly self-reported reasons for using medicinal plants.

**Conclusions:**

Patients with psoriasis in Palestine seem to use medicinal plants as a CAM modality to manage their psoriasis. Many medicinal plants were commonly used by patients with psoriasis. More randomized clinical trials are needed to demonstrate safety and efficacy for the majority of these medicinal plants reported to be used by patients with psoriasis in Palestine.

**Electronic supplementary material:**

The online version of this article (doi:10.1186/s12906-016-1503-4) contains supplementary material, which is available to authorized users.

## Background

Psoriasis is a frequent skin inflammatory disorder that inflicts approximately 2–3% of the populations around the globe [1, 2]. However, prevalence of psoriasis varies across geographical locations and races. Research conducted in the Middle East showed that the incidence of psoriasis in Algeria, Tunisia and Morocco was estimated at 10.36, 13.26 and 15.04 per 1000, respectively [3]. The severity of psoriasis varies from scattered papules to generalized scaly plaques [4]. Depending on location and severity of lesions, psoriasis might have negative consequences on the quality of life of the patients in terms of physical and emotional well-being [4].

Management of psoriasis largely depends on the severity and location of lesions. Systemic therapy, phototherapy and agents applied topically have emerged as therapeutic options in the management of psoriasis [5]. Retinoids, cyclosporine, methotrexate and biologics are approved by the US Food and Drug Administration (FDA) for systemic therapy of psoriasis [6]. The American Academy of Dermatology recommends the use of systemic therapy and phototherapy for sever lesions. Agents applied topically are recommended for mild and localized lesions that do not affect the daily activities of the patient [5]. However, previous studies have shown that systemic therapy and phototherapy are underused because patients prefer topical preparations [7, 8]. Recent statistics showed that only 43% of patients diagnosed with severe psoriasis received systemic therapy [8]. Poor patient adherence and physician reluctance to prescribe systemic treatments were associated with many factors including adverse effects, intolerance, affordability and development of resistance to therapies [1, 9–11].

Published therapeutic guidelines for the management of psoriasis do not fully satisfy the expectations of patients. Moreover, patients with psoriasis experience inevitable avoidance behavior and psychological disturbances that deteriorate their quality of life [1, 12]. Not surprisingly, patients with psoriasis often seek treatments outside the allopathic paradigm to meet their healthcare needs considering the chronic and frustrating nature of the disease [13–15]. Patients with psoriasis were shown to use different modalities of complementary and alternative medicine (CAM) [14, 16]. The use of medicinal plants has evolved as one of the most preferred CAM modalities across different cultures around the globe [13]. Some medicinal plants are used topically while others are ingested for systemic effects. We believe that healthcare professionals should be aware that patients might be using such medicinal plants as an alternative to or in combination with allopathic treatments.

The use of medicinal plants has long been regarded as non-evidence based practice [17]. Today, leading academic and regulatory institutions are setting standards to encourage conducting randomized clinical trials using different CAM modalities including the use of medicinal plants in a scientifically rigorous fashion [18, 19]. A considerable number of randomized controlled clinical trials have been conducted using different medicinal plants to treat signs/symptoms of psoriasis [15]. Some medicinal plants were shown to improve signs and symptoms of psoriasis while other did not shown any significant improvements [14, 15, 17].

As using medicinal plants is based on centuries old traditions, their patterns of use differ from culture to another. Globally, little is known on the association of different sociodemographic factors like age, gender, educational and economic status with the pattern of use of medicinal plants to treat psoriasis. In Palestine, little is known on the use of medicinal plants by patients with psoriasis. The present study was conducted to investigate the pattern of use of medicinal plants among psoriasis patients in Palestine, to assess clinical and sociodemographic predictors of using medicinal plants and to identify perceived benefits from using medicinal plants by patients with psoriasis. The study also aims to identify the sources of information and the underlying reasons for using medicinal plants to treat psoriasis. This study was conducted with special emphasis on the use of medicinal plants as a CAM modality.

## Methods

The present study was undertaken in a cross-sectional observational design at outpatient clinics all over the West Bank of Palestine. A convenient sample was recruited from outpatient clinics in the period of August 2015 to December 2015.

### Structured interview and validation of the questionnaire

The method used in this study was based on our previously published study [13]. Briefly, ten psoriasis patients were interviewed in the first stage of the study to explore their views on the use of medicinal plants to treat their psoriasis and the methods they use in the preparation of these medicinal plants.

A questionnaire containing two sections was developed as in our previous study [13]. In the first section, patients were requested to provide their sociodemographic details such as age, educational status, marital status, place of residence, monthly household income, disease stage, period relapsed since the patient was diagnosed with the disease, type of treatments used, whether the patient used medicinal plants or not (Additional file 1). Inclusion of these sociodemographic variables was based on a literature review of previous studies. In the second section, patients were requested to answer open-ended questions to provide the names of medicinal plants used, reasons why the patient used these medicinal plants, who informed the patient about these medicinal plants, methods of preparing medicinal plants and the sources of these medicinal plants.

The questionnaire was piloted and revised to help understanding. Two trained researchers conducted the interviews and collected the questionnaires from the study participants.

### Statistical analysis

For statistical analysis, patients recruited for this study were categorized as either users of medicinal plants or nonusers based on whether they used medicinal plants or not in the last 6 months. Some data pertaining to period relapsed since the patient was diagnosed with the disease, disease stage and previous treatments were obtained from the medical records of the patients. Pearson’s Chi-Square (*χ* ^2^) or Fisher’s Exact Tests were used to compare categorical groups, as appropriate. Spearman’s rank correlation was used to correlate categorical data. Statistical significance was considered when the *p* value was less than 0.05. Data were processed using IBM SPSS for Windows v.21.0 (IBM-SPSS, Chicago, Illinois, USA). In this analysis, users of medicinal plants were considered equally without regard to the number of medicinal plants they used.

### Ethical approval

The protocol and ethics of the present study were approved by the Institutional Review Board (IRB) of An-Najah National University (protocol No. 62/Aug/2015). Participants signed an informed consent before they took part in the present study. No financial incentives were offered to participants during this study. Participants were assured of their anonymity and all data obtained were kept confidential.

## Results

### Characteristics of participants

In the present study, a total of 149 patients diagnosed with psoriasis were approached and completed the questionnaire. The sociodemographic details of the study patients are shown in Table 1. More than half (53%) of the participants were below 40 years old. About 81 (54.4%) used medicinal plants instead of chemotherapy while the rest of 68 (45.6%) did not used medicinal plants instead of chemotherapy.

**Table 1:** Sociodemographic characteristics of the study patients

Sociodemographic variable	Number of patients	Prevalence (%)
Age (years)		
< 40	79	53.0
≥ 40	70	47.0
Educational status		
No formal education or primary school	35	23.5
Higher education	114	76.5
Monthly household income		
Low	57	38.3
High	92	61.7
Marital status		
Single	44	29.5
Married	100	67.1
Other	5	3.4
Place of residence		
City	62	41.6
Village	79	53.0
Camp	8	5.4
Psoriasis stage		
Early	51	34.2
Late	98	65.8
Period relapsed since diagnosis (years)		
< 2	70	47.0
≥ 2	79	53.0

When the sociodemographic variables of users and nonusers of medicinal plants were compared, characteristics like age and household income were significantly associated as shown by the *χ* ^2^ or Fisher’s exact tests and Spearman’s correlation coefficients (Table 2). There was a significant low correlation between the marital status and use of medicinal plants as shown in Table 2.

**Table 2:** Association of different sociodemographic variables with the use of medicinal plants

Sociodemographic variable	Number of patients	Percent	Users of medicinal plants	Nonuser of medicinal plants	*χ* ^2^ or Fisher’s Exact Test	*P* value	Correlation	*P* value
Age (years)								
< 40	79	53.0	26	53	31.2	<0.001	0.458	<0.01
≥ 40	70	47.0	55	15				
Educational level								
No formal education or primary school	35	23.5	20	15	0.14	0.85	−0.031	0.7
Higher education	114	76.5	61	53				
Monthly household income								
Low	57	38.3	49	8	37.2	<0.001	−0.499	<0.001
High	92	61.7	32	60				
Marital status								
Single	44	29.5	18	26	4.6	0.1	0.169	0.04
Married	100	67.1	60	40				
Other (divorced/widowed)	5	3.4	3	2				
Place of residence								
City	62	41.6	33	29	0.23	0.96	0.012	0.88
Village	79	53.0	44	35				
Camp	8	5.4	4	4				
Psoriasis stage								
Early	51	34.2	25	26	0.89	0.22	0.077	0.35
Late	98	65.8	56	42				
Period relapsed since diagnosis (years)								
< 2	70	47.0	37	33	0.12	0.73	0.028	0.73
≥ 2	79	53.0	44	35				

### Medicinal plants used

In this study, 81 patient with psoriasis declared that they used at least one medicinal plant in the last 6 months to treat signs/symptoms of their psoriasis. The majority of these patients used more than one medicinal plant concurrently.

All patients with psoriasis who participated in this study declared that they used *Aloe vera*. The second most commonly used medicinal herb was *Trigonella arabica* which was reported by 65 (80.2%) patients followed by *Catharanthus roseus* which was reported by 60 (74.1%) and *Anthemis cotula* which was reported by 28 (71.6%). A detailed list of the medicinal plants reported in this study are shown in Table 3.

**Table 3:** Most frequently reported medicinal plants by psoriasis patients in descending order by number of patients in the West Bank of Palestine

Scientific name/voucher number	English common name	Local name	Family	Number of users	Used part	Method of preparation	Application
*Aloe vera* (L.) Burm.f./Pharm-PCT-115	Aloe	Sobar	Xanthorrhoeaceae	35	Leaves	Gel	Aloe leaves are grounded into gel which applied on the affected area once a day.
*Trigonella arabica* Delile/Pharm-PCT-2511	Fenugreek	Helba	Leguminosae	27	Seeds	Paste	Grounded seeds (about 5 table spoons) are mixed with 5 table spoons of Olive oil. This paste is applied on the affected area.
*Catharanthus roseus* (L.) G. Don/Pharm-PCT-2728	Vinca	Wanake	Apocynaceae	25	Entire plant	Paste	Smashed Vinca leaves with Aloe vera gel are mixed. This paste applied on the affected area once a day.
*Anthemis cotula* L./Pharm-PCT-178	Chamomile	Babonaj	Compositae	24	Flowers	Paste	About 50 mL of powdered Chamomile flowers are mixed with 15 mL Olive oil and this paste is applied 3–5 times daily on the affected area.
*Linum usitatissimum* L./Pharm-PCT-2735	Flax	Ketan	Linaceae	15	Seeds	Oil	Oil from Lin seeds is warmed and applied twice a day on the affected area.
*Simmondsia chinensis* (Link) C.K. Schneid./Pharm-PCT-2744	Jojoba	Jojoba	Simmondsiaceae	13	Seeds	Oil	Oil from seeds is applied with extensive massage on the affected area once a day.
*Capsicum annuum* L./Pharm-PCT-2729	Red Pepper	Shatta	Solanaceae	11	Fruits	Ointment	The smashed fruit is mixed with 20 g of Vaseline and rubbed twice a day on the affected area.
*Glycyrrhiza glabra* L./Pharm-PCT-1128	Licorice	Arek alsoos	Leguminosae	10	Roots	Ointment	About 100 g of licorice powder is mixed with 100 mL of Vaseline and applied on the affected area twice a day.
*Persea americana* Mill./Pharm-PCT-2740	Avocado	Avokado	Lauraceae	9	Fruits	Paste	The smashed fruit is mixed with Olive oil and applied on the affected area.
*Ficus carica* L./Pharm-PCT-1028	Fig	Tenn	Moraceae	9	Fruits	Latex	The latex from the fruit is rubbed on the affected area once a day.
*Olea europaea* L./Pharm-PCT-1664	Olive	Zayton	Oleaceae	7	Fruits oil	Oil	Olive oil is applied on the affected area twice a day.
*Nigella arvensis* L./Pharm-PCT-1640	Black Cumin	Kezha	Ranunculaceae	6	Seeds	Oil	Oil from seeds is applied on the affected area twice a day.
*Ammi visnaga* (L.) Lam./Pharm-PCT-139	Khella	Khella masrye	Apiaceae	6	Fruits	Paste	Khella powder is mixed with Olive oil, this paste rubbed on the affected area twice a day.
*Curcuma longa* L./Pharm-PCT-2709	Turmeric	Korkom	Zingiberaceae	6	Rhizomes	Paste	About 250 g of the powdered rhizomes are boiled in 100 mL of Olive oil and the mixture is applied to the affected area once a day.
*Pinus halepensis* Mill./Pharm-PCT-1863	Pine	Sonobar	Pinaceae	5	Wood	Tar	Tar from Pine woods is applied on the affected area twice a day.
*Avena barbata* Pott ex Link/Pharm-PCT-346	Oat	Shofan	Poaceae	5	Seeds	Paste	About 50 g of seed powder is mixed with 25 mL of water and this paste is rubbed on the affected area daily at bed time.
*Juglans regia* L./Pharm-PCT-2714	Walnut	Joz	Juglandaceae	4	Fruits (peels)	Decoction	About 50 g of the leaves are boiled in 100 mL of water and the affected area is washed with this decoction twice a day.
*Prunus amygdalus* var. amara (DC.) Focke/Pharm-PCT-2743	Bitter Almond	Loz mor	Rosaceae	4	Seeds	Oil	About 20 mL of the oil are mixed with black cumin oil and applied on the affecting area once a day along with chamomile tea.
*Malva sylvestris* L./Pharm-PCT-1507	Common mallow	Khobeza	Malvaceae	4	Leaves	Decoction	About 10 g of the leaves are boiled in 100 mL of water. The affected is rubbed with this decoction twice a day.
*Salvia fruticosa* Mill./Pharm-PCT-2117	Sage	Maramya	Lamiaceae	3	Leaves	Decoction	About 20 g of the leaves are boiled in 100 mL of water and the affected is washed with this decoction twice a day.
*Paronychia argentea* Lam./Pharm-PCT-1793	Algerian tea	Rejl alhamama	Caryophyllaceae	3	Entire plant	Paste	About 50 g of the powdered plant are mixed with 100 mL of Olive oil and the affected area is rubbed with this paste once a day.
*Lawsonia inermis* L./Pharm-PCT-2736	Henna	Hena	Lythraceae	3	Leaves	Paste	About 100 g of dried Henna leaves are mixed with 30 mL of water, this paste applied on the affected area once a day.
*Urtica urens* L./Pharm-PCT-2562	Stinging Nettles	Kores	Urticaceae	3	Leaves	Decoction	About 100 g of the leaves are boiled in 100 mL of water and the affected area is washed with this decoction three times a day.
*Senna alexandrina* Mill./Pharm-PCT-2224	Senna	Senamake	Leguminosae	3	Leaves	Paste	About 50 g of dried Senna leaves are mixed with 10 mL of water and this paste is applied topically on the affected area once a day.
*Inula viscosa* (L.) Aiton/Pharm-PCT-2713	Inula	Altayon	Compositae	2	Leaves	Infusion	About 100 g of the leaves are steeped in 100 mL of water for 12 h. The affected area is washed with this infusion twice a day.
*Origanum jordanicum* Danin & Kunne/Pharm-PCT-1729	Thyme	Zaatar	Lamiaceae	2	Leaves	Decoction	About 30 g of the leaves are boiled in 100 mL of water. The affected area is washed with this decoction twice a day.
*Allium sativum* L./Pharm-PCT-2704	Garlic	Thom	Amaryllidaceae	2	Bulb	Vinegar	Garlic bulb vinegar is applied with massage to the affected area twice a day.
*Citrus limon* (L.) Osbeck/Pharm-PCT-2741	Lemon	laemon	Rutaceae	2	Fruits	Juice	About 50 mL of Lemon juice are mixed with 5 mL of concentrated iodine solution. This mixture is applied on the affected area three times a day.
*Zingiber officinale* Roscoe/Pharm-PCT-2724	Ginger	Zangabel	Zingiberaceae	2	Rhizomes	Ointment	About 50 g of the powdered rhizomes are mixed with 50 g of Vaseline and this ointment is rubbed on the affecting area once a day.
*Musa paradisiaca* L./Pharm-PCT-2715	Banana	Moz	Musaceae	2	Fruits	Paste	The smashed fruit peels are rubbed on the affected area twice a day.
*Ricinus communis* L./Pharm-PCT-2742	Castor	Kharwaa	Euphorbiaceae	2	Seeds	Oil	Oil from the seeds are applied on the affected area twice daily.
*Vitis vinifera* L./Pharm-PCT-2665	Grape	A’nab	Vitaceae	2	Fruits	Vinegar	The affected area is washed three times a day with Grape vinegar.
*Teucrium capitatum* L./Pharm-PCT-2407	Germander	Ja’ade	Lamiaceae	2	Leaves	Decoction	About 50 g of the leaves are boiled in 100 mL of water and the affected area is bathed with this decoction once a day.

Names of plant families in this table were obtained from The Plant List (http://www.theplantlist.org)

Leaves and fruits were the most commonly used parts of the medicinal plants. The rest of parts used by the study patients are shown in Table 3. Medicinal plants were most frequently prepared in the form of pastes (Table 3). Medicinal plants were obtained from different sources. The majority of the patients obtained their medicinal plants from wild life (Table 4). When asked about their source of knowledge on medicinal plants, the majority of the study participants stated that medicinal plants were recommended by other patients with psoriasis (Table 4).

**Table 4:** Sources, knowledge and reasons for using or not using medicinal plants

Item	Percent
Sources from where patients obtained their medicinal plants	
Wild life	47.9
Herbalists	20.5
Friends	12.3
Pharmacists	1.4
Undeclared	17.8
Sources from where patients obtained knowledge about medicinal plants	
Family friend	8.33
Other patients	31.5
Herbalist	25.9
Public media	16.7
Pharmacists	0.93
Doctors	1.85
Internet	14.8
Reason reported by patients for using medicinal plants	
Medicinal plants enhance my immune system	16.7
I was advised to use medicinal plants	15.8
Medicinal plants are available and affordable	5
Using medicinal plants is safe	27.5
Medicinal plants are effective	11.7
I have sufficient experience and information about medicinal plants	6.67
Medicinal plants improve conventional therapy and reduce their side effects	16.7
Reason reported by patients for not using medicinal plants	
I don’t have enough information on medicinal plants	28
I am not convinced that medicinal plants are better than conventional therapies	16
Medicinal plants might have serious side effects	56

### Reasons for using or not using medicinal plants

When asked why they use medicinal plants to manage their psoriasis, patients using medicinal plants gave various reasons. The most commonly reported reason was safety. Other reasons reported in this study are shown in Table 4.

When nonusers were asked why they were not using medicinal plants to manage their psoriasis, again they mentioned many reasons of which concerns over safety was the most frequently reported reason. Other reasons are shown in Table 4.

## Discussion

This is the first investigation on the use of medicinal plants in patients with psoriasis in Palestine. The findings from this study showed that 54.4% of the patients included in this study used medicinal plants to treat signs/symptoms of their psoriasis. Our results were consistent with prior studies in which the prevalence of CAM use was in the range of 43–69% in different countries including the US, UK and Taiwan [15, 20–22].

Comparisons using *χ* ^2^ or Fisher’s exact analyses showed that users of medicinal plants differed with regard to age and monthly household income. However, educational level, place of residence, stage of the disease and time relapsed since diagnosis were not significantly associated with the use of medicinal plants. Nonparametric correlation showed that age and marital status were significantly and positively correlated with the use of medicinal plants. However, monthly household income was significantly and negatively correlated with the use of medicinal plants. In this study, the most commonly reason for using medicinal plants was the belief that these CAM modalities were safe (27.5%). Patients also reported enhancing immunity and reducing side effects of conventional therapies (16.7%). These results were in line with those reported in the UK in which dissatisfaction with the results of conventional therapies was the most commonly reported reason for using CAM modalities (including the use of medicinal plants) by patients with psoriasis [23].

This study showed that 33 different plants were used with various methods of preparation by patients to treat their psoriasis. The most commonly used plants were *Aloe vera*, *Trigonella arabica*, *Catharanthus roseus* and *Anthemis cotula*. Some results were not surprising as *Aloe vera* was reported to be commonly used by patients with psoriasis in prior studies [15, 17, 24, 25]. Randomized clinical trials were conducted using a cream containing *Aloe vera* in patients with psoriasis which showed statistically significant improvements [24]. Progressive reduction of lesions, desquamation, decreased erythema, infiltration, lower psoriasis area and severity index score (PASI) were considered as improvements [24]. PASI scores in the treatment group decreased from 9.7 to 2.2 compared to 8.9 to 8.2 in the placebo-controlled group [24]. Untoward effects were not reported in this study. Paulsen et al. used a gel containing *Aloe vera* in a randomized placebo-controlled trails [25]. Modified PASI scores decreased in 75.2% of patients in the treatment group compared to placebo group [25]. Probably, patients in our study witness improvements from using *Aloe vera* based preparations and spread the word to family, friends and other patients. Interestingly in this study, a significant number of patients with psoriasis learned about medicinal plants from family friends and other patients with psoriasis.

Many of the plants reported by the patients in this study were used for their promised activity against psoriasis [26]. The rhizomes of *Curcuma longa* has been in use in traditional medicine since thousands of years to treat various inflammatory disorders including psoriasis [26]. The main constituent in *Curcuma longa* is curcumin to which anti-psoriatic activities are attributed. It is thought that curcumin targets various transcription factors such as NF-KB, AP-1 and PPARγ, enzymes such as COX2, 5-LOX, iNOS and hemeoxygenase- 1, cell cycle proteins such as cyclin D1 and p21, cytokines like TNF, IL-1, IL-6 and some cell surface adhesion molecules [26–28]. *Zingiber officinale* is also thought to exhibit anti-psoriatic activity though 6-Gingerol which is a natural analogue of curcumin [29]. *Glycyrrhiza glabra* was shown to possess anti-proliferative and anti-inflammatory activities in psoriasis [30]. The use of *Glycyrrhiza glabra* was reported to be safe, effective and inexpensive [26, 30].

Some of the medicinal plants used by the patients who participated in this study were not previously reported to be used to treat psoriasis. These plants include *Trigonella arabica*. However, other members of the same leguminosae family like *Psoralea corylifolia* L. were shown to contain phenolic glycosides which might inhibit keratinocytes replication in psoriasis [31]. *Crotalaria emarginella* Vatke is another member of the same family was shown to contain crotalic acid which exhibited anti-inflammatory action in psoriasis [26].

Interestingly, many of the medicinal plants reported to be used in this study are edible and widely used in folk diet. This might suggest that these plants could be nontoxic or possess low toxic activities. However, it is important not to overestimate the safety and efficacy of the medicinal plants reported to be used in ethnopharmacological surveys. Prior studies showed that some of the medicinal plants used were associated with unwanted side effects. For example, some *Trigonella arabica* species were reported to have hypoglycemic effect when used systemically [32]. However, it is noteworthy to mention that topical application on the skin is often associated with less severe side effects than systemic exposure.

It is also important to note that the efficacy of some treatments were questionable as randomized clinical trials failed to demonstrate significant improvements in psoriasis patients [15, 17]. For example, Stucker et al. treated 13 patients with psoriasis topically with a combination of vitamin B12 and avocado oil, after 12 weeks no statistically significant reduction in PASI scores was observed [33].

As *Aloe vera*, *Trigonella arabica*, *Catharanthus roseus* and *Anthemis cotula* were the most frequently used medicinal plants by patients with psoriasis who participated in this study, a brief review of their uses, side effects, in vivo and in vitro activity in psoriasis are briefly reviewed and summarized in Table 5.

**Table 5:** Summary of published ethnopharmacological uses, side effects, in vivo and in vitro activity of the most frequently used plants against psoriasis

Plant species	Reported ethnopharmacological use with reference source	In vivo *and* in vitro *activity against* psoriasis with reference source	Side effects and toxicity with reference source
*Aloe vera*	The plant was used in folk medicine for the treatment of psoriasis in South Africa [34], Turkey [35], Pakistan [36], India [37], Middle East [38], Palestine [17, 39] and Mexico [40].	Hydrophilic cream and gel of *Aloe vera* were tried and showed significant improvement against psoriasis in a randomized clinical trial [24, 25]. In another study, topical *Aloe vera* with 0.1% triamcinolone acetonide was used in mild to moderate plaque psoriasis. *Aloe* showed its ability to reduce the clinical symptoms of psoriasis [41]. Aloe extract showed anti-psoriatic activity of 81.95%, compared with 87.94 for tazarotene in the mouse tail model of psoriasis [42].	Localized side effects including dryness, stinging, soreness, fissures, erythema and contact urticaria may develop after using topical Aloe [25, 43, 44].
*Trigonella arabica*	The plant was used in ethnomedicine for treatment of psoriasis in Palestine [45] and Pakistan [46].	No reference	No reference
*Catharanthus roseus*	No reference	*C. roseus* reduced the expression of psoriatic marker, keratin 17 (K17) in human keratinocytes [47].	No reference
*Anthemis cotula*	No reference	No reference	Contact sensitization [48].

In the present study, the majority of the medicinal plants were prepared in the form of pastes. Pastes were most commonly prepared from leaves and fruits. This was consistent with the nature of the disease and the intended action as agents were meant to be applied topically. Taken together, a considerable percentage of patients who used medicinal plants believed in their safety and therapeutic power. This was consistent with our recent study in which patients with cancer who used medicinal plants believed in their power to treat breast cancer in the West Bank [13].

## Conclusions

The prevalence of use of medicinal plants among psoriasis patients in Palestine was not known. This study showed that the use of medicinal plants was prevalent among patients with psoriasis in Palestine. This ethnopharmacological survey showed that *Aloe vera*, *Trigonella arabica*, *Catharanthus roseus* and *Anthemis cotula* were the most frequently used medicinal plants to treat psoriasis. The use of medicinal plants was associated with age and household income of the patients. The majority of the patients using medicinal plants believed in their power of treatment and safety. More randomized clinical trials are needed to demonstrate the efficacy and safety of these medicinal plants in patients with psoriasis.

## Additional file

Additional file 1: Interviewer administered survey of medicinal plants use by psoriasis patients in the West Bank of Palestine. (DOCX 18 kb)

## Abbreviations

CAM: Complementary and alternative medicine
IRB: Institutional Review Board
PASI: Psoriasis area and severity index
