# Supplementary material for: Ethnopharmacological survey of medicinal plants used by patients with psoriasis in the West Bank of Palestine
Source: BMC Complement Altern Med. 2017 Jan 3;17:4. doi: 10.1186/s12906-016-1503-4 (PMC5209870; doi:10.1186/s12906-016-1503-4)
Supplement: Additional file 1: — Interviewer administered survey of medicinal plants use by psoriasis patients in the West Bank of Palestine. (DOCX 18 kb) [file 12906_2016_1503_MOESM1_ESM.docx]

**Interviewer administered survey of medicinal plants use by psoriasis patients in the West Bank of Palestine**

| **Gender** | | | | |
| --- | --- | --- | --- | --- |
| - Male | | | | |
| - Female | | | | |
| **Age (years)** | | | | |
| - <40 | | | | |
| - ≥40 | | | | |
| **Educational status** | | | | |
| - No formal education or primary school | | | | |
| - Higher education | | | | |
| **Monthly household income in Jordanian Dinars (JDs)***  * Low : <750 JDs; ≥ 750 JDs | | | | |
| - Low | | | | |
| - High | | | | |
| **Marital status** | | | | |
| - Single | | | | |
| - Married | | | | |
| - Other | | | | |
| **Place of residence** | | | | |
| - City | | | | |
| - Village | | | | |
| - Camp | | | | |
| **Psoriasis stage** | | | | |
| - Early | | | | |
| - Late | | | | |
| **Period relapsed since diagnosis (years)** | | | | |
| - <2 | | | | |
| - ≥2 | | | | |
| **Do you use medicinal plants to treat your psoriasis?** | | | | |
| - Yes | | | | |
| - No | | | | |
| **In case you use medicinal plants to treat your psoriasis, please mention:** | | | | |
| # | Name of the  plant used | Part of the plant used | Method of preparation | Method of application |
| 1 |  |  |  |  |
| 2 |  |  |  |  |
| 3 |  |  |  |  |
| 4 |  |  |  |  |
| 5 |  |  |  |  |
| 6 |  |  |  |  |
| **From where do you obtain your medicinal plants?** | | | | |
| - Wild life | | | | |
| - Herbalists | | | | |
| - Friends | | | | |
| - Pharmacists | | | | |
| - Others, please specify: …………….. | | | | |
| **From where did you learn about medicinal plants?** | | | | |
| - Family friend | | | | |
| - Other patients | | | | |
| - Herbalists | | | | |
| - Public media | | | | |
| - Pharmacists | | | | |
| - Doctors | | | | |
| - Internet | | | | |
| **Why do you use medicinal plants?** | | | | |
| - Medicinal plants enhance immunity | | | | |
| - I was advised to use medicinal plants | | | | |
| - Medicinal plants are available and affordable | | | | |
| - Use of medicinal plants is safe | | | | |
| - Medicinal plants are effective | | | | |
| - I have sufficient experience and information about herbal remedies | | | | |
| - Medicinal plants improve conventional therapy and reduce side effects | | | | |
| In case you do not use medicinal plants, please mention why? | | | | |
| - I don't have enough information on medicinal plants | | | | |
| - I am not convinced that medicinal plants are better than drugs | | | | |
| - Medicinal plants might have serious side effects | | | | |
